# The role of quadriceps muscle strength in the development of falls in the elderly people, a cross-sectional study

**DOI:** 10.1186/s12998-018-0195-x

**Published:** 2018-08-06

**Authors:** Alijan Ahmadiahangar, Yahya Javadian, Mansour Babaei, Behzad Heidari, Seyedreza Hosseini, Mohammad Aminzadeh

**Affiliations:** 1 Mobility Impairement Research Center, Health Research Institute, Babol University of Medical Sciences, Babol, I.R. Iran, Ganjafrouz Ave., Babol, 4717641367 Iran; 2 Department of Physiotherapy, School of Rehabilitation, Babol University of Medical Sciences, Babol, I.R Iran, Ganjafrouz Ave., Babol, 4717641367 Iran; 3 Clinical Research Development Unite of Rouhani Hospital, Babol University of Medical Sciences, Babol, I.R. Iran, Ganjafrouz Ave., Babol, 4717641367 Iran; 4 Social Determinants of Health Research Center, Health Research Institute, Babol University of Medical Sciences, Babol, I.R.Iran, Ganjafrouz Ave., Babol, 4717641367 Iran; 5 School of Medicine, Babol University of Medical Sciences, Ganjafrouz Ave., Babol, 4717641367 Iran

**Keywords:** Elderly people, Fall, Quadriceps muscle strength

## Abstract

**Background:**

Falls are a major health issue in the elderly people and an important cause of bone fracture. The aim of this study was to determine the association between quadriceps muscle strength (QMS) and falls in the elderly subjects.

**Methods:**

All eligible participants of the Amirkola Cohort Study entered the study. Data regarding demographic characteristics, clinical and laboratory examinations were provided between 2011 to 2014. Occurrence of falls during previous year was determined by interview and review of the medical records. The study patients were divided into low, moderate and high muscle strength groups according to QMS values ≥ 30, 15–30, and < 15 kg respectively). Association between muscle strength and falls was determined by using multiple logistic regression analysis with calculation of odds ratio (OR).

**Results:**

A total 1028 participants (females, 44.3%) were analyzed and 178(17.3%) subjects experienced a fall. Individuals with falls had higher age (*p* = 0.001) and lower QMS value (*p* = 0.001). After adjustment for all clinical and demographic variables, occurrence of falls was negatively associated with QMS and positively associated with age > 70 years old. Compared to group with QMS ≥ 30 kg, the prevalence of falls in low and moderate QMS groups increased by OR = 3(95% CI, 1.78–5.05) and 2.18 (95% CI, 1.22–3.42) respectively.

**Conclusion:**

These findings indicate that older subjects with lower QMS are at greater risk of falls. These findings provide a rational for muscle strengthening exercise in older people.

## Background

Aging is associated with several comorbidities, which alter physiological and anatomical status of all organs including muscle strength [1].By aging, reduction of muscle mass and strength result in decreased in mechanical properties of the skeletal muscles [2]. Falls is a major health issue in the elderly people and an important cause of bone fractures, disability, and mortality. Development of falls can be prevented by appropriate intervention targeting relevant associated factors. It has been reported that at least one third of the elderly people will experience fall in a year, and about two-thirds of patients who had a fall attack, are likely to experience another fall in the following 6 months [3]. Around 40% of the falls in the elderly individuals aged > 85 years old lead to fatality, while the remaining 60% will succumb to expensive diagnostic care and treatment procedures with long period of hospitalization [4]. Apart from the physical damages of the fall, elderly individuals with a history of fall would often experience loss of self-confidence due to fear of subsequent attacks as well as inability to perform daily tasks. This issue leads to depression and dissociation from society [5].

Most of the falls are associated with one or more identifiable risk factors [6, 7].Recognition of the modifiable factors can be helpful in preventing ensuing morbidity, mortality and economic costs [5, 6]. Muscle weakness, impaired balance and unsteady gait are usually precritical signs and symptoms of fall [6, 7], and is usually a common finding among the aged population, which have been consistently recognized as a risk factor for development of falls and related injuries [8].Case–control studies have demonstrated higher than expected risk of falls and fractures among individuals with gait and muscle dysfunctions [7].Relationship between muscle strength and falls has been shown in many previous studies [6, 9–12], and the effect of muscle strengthen exercises in reducing falls and subsequent disability have been also shown [8, 11, 13]. Nonetheless, data regarding the contributive role of quadriceps muscle in the development of falls in the elderly subjects are scarce.

A positive association has been observed between the quadriceps muscle activity and stability as well as gait [14]. Quadriceps muscle weakness is an important risk factors in the development of knee osteoarthritis and disability in the elderly populations [15, 16].Reduction of quadriceps muscle strength by aging, imposes the elderly people at greater risk of disability and falls [5, 15, 17, 18] whereas, raising muscle power offers a new opportunity to reduce the risk of falls and fall-related injuries. We hypothesized an association between quadriceps muscle strength and falls in the elderly people, and thus the present cross-sectional case-control study was designed to determine the relationship between quadriceps muscle strength (QMS) and falls in an elderly cohort of people aged 60 years and more.

## Methods

The patients of this cross-sectional study were recruited among the participants of the Amirkola Health and Ageing Project (AHAP). This project was carried out in the north of Iran, located in Southern region of the Caspian Sea shores. The project was conducted for investigation of the geriatric medical problems such as falling, bone fragility and fractures, cognitive impairment with dementia, poor mobility and functional dependence. This study was approved by the Ethics Committee of the Babol University of Medical Sciences, Babol, Iran (ethic cod: mubabol.rec.1393.12).

All inhabitants aged 60 years and over were invited to participate in this study and finally 72.3% of the invited subjects participated in the project and a total of 1028 eligible participants were analysed. Data were provided between 2011 to 2014 in regard to demographic characteristics such as age, sex, educational level, physical activity, lifestyle, biochemical characteristics, medications, and the presence of comorbidities as well as history of falls. Details of patients’ selection, data collection, validity of the questionnaires have been described elsewhere [19]. In brief, the demographic data, physical activity score, the presence of medical conditions, drugs history were provided by interview and fill in questionnaires as well as clinical examination. Diagnoses of each condition was confirmed by relevant criteria [19] Occurrence of falls as well as the time of fall occurrences was confirmed by interview based on self-reported data and review of the medical records. The QMS of both lower limbs was assessed by a single experienced physiotherapist using dynamometry methods [20]. In this method the patient is seated on a chair and the dynamometer at one end is fixed 5 cm above the lateral malleolus of the tibia and at the other end is fixed to the wall. The knee joint is at 90 degree of flexion. The patient extends the knee joint (concentric contraction of the quadriceps muscle). The quadriceps muscle strength will be measured at the maximum of the quadriceps muscle contraction by dynamometer. The average value of three measurements was considered for analysis. The reliability of QMS measurements was confirmed by test- retest reliability method in 20 consecutive patients in whom QMS measurement was repeated after 30 min rest. The correlation coefficient value between the two sets of QMS scores was 0.97 (*P* = 0.001). The validity of dynamometer was tested by known specified metal weights of 5-kg, 10 -kg, and 20-kg at the beginning of the study and thereafter during the study periods [18].

Exclusion criteria were history of stroke, the presence of inflammatory or non-inflammatory musculoskeletal disorders particularly dependent individuals with advanced osteoarthritis, peripheral neuropathies, and the presence of comorbidities like congestive heart failure, chronic lung diseases and lower limbs fractures. Distributions of all variables were determined by measures of skewness and kurtosis. Normality of distribution was assessed by using Kolmogrov-Smirnov test. Parametric tests were used for comparison of variables with normal distribution and non-parametric tests for variables with skewed distributions.

In statistical analyses, the participants were classified according to quadriceps muscle strength as high (QMS > 30 kg), moderate (15–30 kg) and low (< 15 kg) group. The prevalence of patients with falls in the moderate and low QMS groups was compared with the QMS ≥ 30 kg as the control group. Chi square test with calculation of odds ratio (OR) and 95% confidence interval (95%CI) was used to determine the association. Multiple regression analysis with simultaneous adjustment for all covariates was applied to determine independent association. All analyses were performed by SPSS version 18.

## Results

A total of 1028 participants (women, 44.3%) were analyzed. The demographic characteristics and the distribution of all variables are shown in Table 1. Overall, 178 participants (17.3%) had experienced at least one fall in the past year. Mean age in subjects with history of falls was significantly higher (69.8 ± 7.7 vs 67.9 ± 7.7 years old, *p* = 0.001)), and the value of QMS was significantly lower (20.13 ± 9.3 vs 23.6 ± 10.6 kg, *p* = 0.001) as compared with those without falls. As shown in Table 1, subjects with and without falls were different only in regard to mean age, proportion of female gender, osteoporosis and QMS values. The crude and the adjusted odds of the association are presented in Table 2. There was an inverse dose-response pattern of relationship between fall and QMS. Compared to group with QMS values ≥ 30 kg as the control group, the prevalence of fall increased by OR = 3 (95%CI, 1.78–5.05, *p* = 0.001) in patients with QMS < 15 kg, and by OR = 2.18 (95% CI, 1.22–3.42, *p* = 0.002 in patients with QMS values between 15 to 30 kg (Table 2). The odds of fall in women was significantly higher as compared with men (21.1% versus 14.3%), OR = 1.60 (95% CI, 1.16–2.22).

**Table 1:** Characteristics of the study elderly population aged 60 years and older with and without falls

Variables	Subjects with falls(*n* = 178) Mean ± SD	Subjects without falls(*n* = 850) Mean ± SD	*P* values
Age, year	69.8 ± 7.7	68 ± 6.9	0.001
Gender			
Women	96 (21.1)	359 (78.9)	0.043
Men	82 (14.3)	491 (85.7)	–
Vitamin D ng/ml	38.1 ± 37	34 ± 31	0.17
Total PA^a^ score, mean ± SD	113 ± 68	115.4 ± 63	0.67
Alcohol consumption			
No	176 (98.88)	820(96.5)	0.09
yes	2 (1.2)	30(3.5)	
Visual impairment, no(%)			
Yes	79 (44.3)	308(36.2)	0.17
No	99 (55.7)	542(63.8)	–
Osteoporosis ^b^			
Yes	83 (46.6)	276 (32.4)	0.001
No	95 (53.4)	574(61.6)	–
Vitamin D deficiency, no(%)			
Yes	66 (37)	311 (63)	0.93
No	112 (62)	539(38)	–
Diabetes			
Yes	59 (33.1)	252 (29.6)	0.50
No	119 (66.8)	598 (70.3)	–
Hypertension, no(%)			
Yes	112 (62.)	518 (61)	0.80
No	66 (37)	332 (63)	–
Obesity ^c^, no(%)			
Yes	62 (34.8)	270 (31.7)	0.57
No	106 (65.2)	580 (68.2)	–
QMS ^d^, kg			
> 30	23 (9.30)	225 (90.7)	–
15–30	97 (18.2)	430 (81.8)	0.001
< 15	58 (23.5)	189 (76.5)	0.001

^a^Physical activity was assessed by administration of the Physical Activity Scale for the Elderly (PASE) questionnaire [42]

^b^Defined as BMD T-score ≤ − 2.5

^c^Defined as body mass index > 30 kg m^2^

^d^Quadriceps muscle strength

**Table 2:** Asoociated factors of fall in the elderly participants aged 60 years and more, with calculation of crude odds ratio (OR) with 95% confidence interval (95% CI) and adjusted OR using multiple regression analysis

Variable	Subject with falls *n* = 178	Subject without falls *n* = 850	Crude OR (95%CI)	Adjusted OR (95%CI)
Gender n(%)				
Men	82 (14.3)	491 (85.7)	1	1.15(0.74–1.79)
Women	96 (21.1)	359 (78.9)	1.6(1.16–2.22)	1
^a^BMI(kg/m^2^)				
> 30	62 (18.7)	270 (81.3)	1	1
25–30	74 (17.2)	357 (82.8)	1.11(076–1.6)	1.09(0.68–1.7)
< 30	42 (15.8)	223 (82.4)	0.82(053–1.26)	1.1(0.71–0.69)
^b^Osteoporosis n(%)				
Absent	95 (14.2)	574 (85.8)	1	1
Present	83 (23.1)	276 (76.9)	1.88(1.34–3.52)	1.38(0.78–1.9)
^c^QMS (kg)				
> 30	23 (9.3)	225 (90.7)	1	1
15–30	97 (18.2)	430 (81.8)	2.18(1.34–3.52)	1.72(1.01–2.9)
< 15	58 (23.5)	189 (76.5)	3.01(1.78–5)	2.02(1.06–3.85)

^a^Body mass index

^b^Defined as BMD T-score ≤ − 2.5

^c^Quadriceps muscle strength

Prevalence of falls was significantly higher in subjects aged > 70 years as compared with those ≤70 years, OR = 1.7(95% CI, 1.23–2.36). In osteoporotic subjects, the prevalence of falls was significantly higher OR = 1.88(95%CI, 1.32–2.52).Prevalence of falls in obese individuals was higher than normal weight subjects but the difference did not reach to a significant level (26.7% vs 15.8%, OR = 1.22, 95% CI, 0.79–1.87).

In multivariate logistic regression analysis, after controlling for all medical covariates (Table 1), a significant association was observed only between falls with QMS as well as age > 70 years old. There was no association between falls and other factors such as vitamin D deficiency, diabetes, visual impairment, hypertension, cerebrovascular disease.

## Discussion

The findings of this study indicate that a significant proportion of elderly subjects aged 60 years and more may experience an incident fall over the previous year. Weakness of quadriceps muscles and age > 70 years were independently associated with falls.

Occurrence of falls in the elderly subjects are common and its prevention is important because, falls account for 87% of all osteoporotic fractures in the elderly [6] and fall-related injuries is associated with significant economic costs suggesting effective preventive strategies to decrease the incidence and healthcare expense of these injuries [5].

The results of a systematic review of the relevant studies showed that, impaired balance and gait, polypharmacy, history of previous falls, advancing age, female gender, visual impairments, cognitive decline especially attention and executive dysfunction, and environmental factors were the major risk factors of falls in older adults [6].

A systematic review and meta-analysis of 30 studies demonstrated that muscle weakness especially lower limbs muscle weakness were the most important factor of occurrence and recurrences of falls [21]. Another systematic review of 21 studies, which have addressed the incidence and risk factors of falls in the Chines older people showed, fall rates between 14.7 and 34%. The associated factors were female sex, older age, use of multiple medications, gait instability, fear of falling, and decline in activities of daily living [22].

However, depending on the study design, duration of follow-up and characteristics of the study population, the prevalence and the causes of falls vary across diverse studies and changes from 13 to 64.5% across different studies [3, 23–28].

The results of this study indicate a significantly negative association of QMS with the development of incident falls. The prevalence of falls in these patients increased from 9.3% in individuals with QMS > 30 kg to 23.5% in those with QMS lower than 15 kg. These findings are in agreement with the results of other studies [1, 9, 12, 21, 23, 29, 30]. In a study by Ikoez et al., 18% of falls have been attributed to quadriceps muscle weakness [9]. In another prospective 3-year longitudinal study, quadriceps muscle strength was predictor incident falls amongst community-dwelling older women at high risk of fracture [10].

These observations indicate a potential role for QMS in predicting future development of falls in older people. Higher risk of falls in older age groups of this study should be also attributed to muscle weakness due to aging. By aging the ability of muscle to regeneration, repair and remodeling diminishes, which results in age-related loss of skeletal mass and strength. In addition, muscle degeneration in older subjects reduces muscle power and mass. These changes may affect maintaining body balance [29, 31]. Although the association of female sex with falls in this study diminished to no significant level but other studies have reported higher prevalence of falls in women [23–26]. Women may be more susceptible to falls, because, several associated factors of falls such as osteoporosis, osteoarthritis, obesity and vitamin D deficiency are more prevalent in women [15, 32–35]. Lack of association in this study may be explained by high frequency of these factors in the control group. Therefore, a significant association might be detected in a study with larger sample size.

In this study, the association of several factors such as obesity, osteoporosis, vitamin D deficiency, hypertension and visual impairment with fall did not reach to a statistical level (Table 1). Vitamin D deficiency affect antigravity effects of lower limb muscles which are responsible for postural balance and correction of deficiency may decrease the occurrence of falls in the elderly [36]. Vitamin D deficiency is associated with QMS as well as knee osteoarthritis. Normalization of vitamin D improves quadriceps muscle strength [15, 18, 20, 37–39].The relationship between knee or hip osteoarthritis as well as obesity with increased risk of falls has been shown [40, 41]. However, lack of association in this study should be explained by high prevalence of these factors in the general population. A study with larger sample would be required to confirm these findings.

This study has limitations regarding the study design which is cross-sectional and the observed association does not indicate causality. Another limitation of this study may be attributed to the method of data collection in respect to incident falls, which was based on self-reported data. It is possible that a proportion of elderly patients could not remember past occurrence of falls, because of cognitive or attention impairment, and the prevalence of falls being underestimated.

The strength of study is based on patient selection which comprised all eligible participants of the Amikola Cohort Study. Collection of data by using validated questionnaire. Furthermore, complete clinical and laboratory examinations of all participants should be also considered strength.

## Conclusion

The findings of this study indicate occurrence of falls in substantial proportion of the elderly people aged 60 years and older. Prevalence of falls was negatively associated with QMS strength, and increased after 70 years old. This issue indicates that elderly people with higher QMS are at lower risk of falls, suggesting a beneficial preventive effect of muscle strengthening. This issue requires to be confirmed in a prospective study.
